# Can Erythropoietin Open a Novel Avenue for Periodontal Regeneration?

**DOI:** 10.7759/cureus.52825

**Published:** 2024-01-23

**Authors:** Meran Farid, Fatma Ata, Eman S Elhennawy, Jilan Youssef

**Affiliations:** 1 Department of Periodontology and Oral Medicine, Faculty of Dentistry, Horus University, Demiatta, EGY; 2 Department of Oral Medicine, Periodontology, Diagnosis, and Oral Radiology, Faculty of Dentistry, Mansoura University, Mansoura, EGY; 3 Department of Clinical Pathology, Faculty of Medicine, Mansoura University, Mansoura, EGY

**Keywords:** clinical attachment level (cal), scaling and root planing, enzyme-linked immunosorbent assay (elisa), probing depth, periodontitis, local drug delivery, clinical attachment level

## Abstract

Introduction: Periodontitis is a dramatic inflammatory disease, representing vigorous interactions between specific causative pathogens and host immune responses resulting in the activation of the destructive inflammatory cascade with the subsequent irreversible destruction of the teeth-supporting apparatus.

Aim: This study aims to evaluate the effect of using erythropoietin (EPO) injectable hydrogel, as an additional therapeutic option to scaling and root planing (SRP) in the treatment of stage II periodontitis patients, and to assess its effect on the level of osteocalcin and interleukin (IL)-1β in the gingival crevicular fluid (GCF).

Methodology: A total number of 40 patients clinically diagnosed with stage II periodontitis were included. The participants were allocated into two equal groups: study and control groups. Patients in the control group received SRP, while those in the study group received SRP followed by injectable hydrogel containing EPO. Clinical parameters such as plaque index (PI), gingival index (GI), probing pocket depth (PPD), and clinical attachment level (CAL) were assessed at baseline and two months post treatment. GCF samples were collected at baseline and two months post treatment from both groups to analyze GCF IL-1β and osteocalcin levels using enzyme-linked immunosorbent assay (ELISA).

Results: Significant reductions in all tested clinical parameters were revealed in both groups in comparison to baseline values. A marked significant reduction in GCF IL-1β level was detected in the study group. However, two months post treatment, the osteocalcin level was decreased significantly in both groups.

Conclusion: This preliminary study shows great promise for the local application of EPO hydrogel as an adjunct to SRP for the management of stage II periodontitis.

## Introduction

Periodontitis is a dramatic inflammatory disease, representing vigorous interactions between specific causative pathogens and host immune responses resulting in the activation of the destructive inflammatory cascade with the subsequent irreversible destruction of the teeth-supporting apparatus [1]. The treatment of periodontitis emphasizes on striking the destructive series by eradicating the pathogen load in the diseased site. The conventional non-surgical periodontal therapy (NSPT) is regarded as the gold standard periodontal therapy. Even though deep and tortuous periodontal defects are inaccessible to be reached by conventional modalities, periodontopathic bacteria may lick into those niches [2]. To minimize the possibility of bacterial recolonization resulting from the conventional therapy limitations, additional therapeutic agents have been used complementary with scaling and root planing (SRP) but unfortunately have some unwanted effects. Local drug delivery (LDD) systems containing antibiotics, antiseptics, herbal products, and/or osteoregenerative agents have been widely used targeting the diseased site at efficacious concentrations with no systemic effect [3-5].

Erythropoietin (EPO) is a highly acting glycoprotein cytokine over a broad range of the body organs and tissues, exerting hematopoietic and non-hematopoietic effects including anti-inflammatory, antioxidative, osteogenic, angiogenic, and anti-apoptosis functions [6]. It exerts its anti-inflammatory effect via the downregulation of pro-inflammatory cytokines, such as interleukin (IL)-1β, IL-6, IL-8, and TNF-α, and by inducing the expression of anti-inflammatory cytokines, transforming growth factor beta (TGF-β), and IL-10 and arginase-1 (Arg-1) helping in the recovery of the periodontium [7,8]. It has been proved that EPO has control over the level of reactive oxygen species (ROS). EPO can alter the polarization of macrophages from M1 to M2. These series result in a decreased level of matrix metalloproteinase-9 (MMP-9) and cyclooxygenase-2 (COX-2). Hence, EPO favors periodontal healing by restoring homeostasis [8].

EPO osteogenic ability is clarified via direct and indirect pathways. EPO promotes bone formation directly by the stimulation of osteoblastic differentiation. Indirect EPO bone regenerative action could be carried out by different pathways. One of them is increasing the expression of bone morphogenetic protein-2 (BMP-2) [9]. Another pathway is EPO increases the expression of runt-related transcription factor (RUNX2), a bone-forming marker displayed at the end of osteoblastic differentiation promoting the expression of the bone morphogenic protein. Additionally, EPO was found to mediate bone homeostasis through EphrinB2/EphB4 inducing osteoblastic differentiation and osteoclastic suppression, resulting in bone formation [10]. Hence, EPO can stimulate osteoblastic proliferation and inhibit the osteoclastic resorptive function [11].

EPO exhibits an angiogenic capacity which results in increasing vascular endothelial growth factor (VEGF) expression with a resultant increase in the vascular density and extracellular matrix maturation favoring the bone regeneration by providing the proliferating and differentiating cells with sufficient oxygen levels as cell nutrition is a must to ensure bone reconstruction [12].

IL-1β is a pro-inflammatory cytokine expressed particularly by many immune cells such as macrophages, natural killer (NK) cells, monocytes, and neutrophils. It is a strategic cytokine in the periodontal pathogenesis. It is also responsible for increased local blood flow and the recruitment of leukocytes. IL-1β also triggers other inflammatory mediators' activation such as IL-6, prostaglandin, and MMPs. IL-1β along with other cytokines promotes the differentiation and activation of osteoclasts. It increases the expression and activation of MMPs, stimulating the degradation of the extracellular matrix and, in turn, leading to tissue destruction and bone resorption [13]. 

Osteocalcin is an osteogenic marker expressed by osteoblasts, odontoblasts, and hypertrophic chondrocytes. It has a double possession in both bone resorption and mineralization. Osteocalcin is regarded as a bone formation biomarker although high levels may be detected during the bone remodeling process. In the conditions exhibiting increased rate of bone turnover such as fracture repair, osteoporosis, and multiple myeloma, elevated levels of serum osteocalcin have been recognized [14]. 

Possessing multifunctional properties, EPO has attracted recently increasing attention for the development of new avenues for periodontal tissue regeneration. This research intended to evaluate the efficacy of using EPO complementary with SRP in the treatment of periodontitis.

## Materials and methods

Participants

This research was performed on 40 systemically healthy patients of both genders, aged between 25 and 55 years. They were selected from the outpatient clinic of the Department of Oral Medicine and Periodontology, Faculty of Dentistry, Mansoura University. By the 2017 classification of periodontal diseases, they were identified as having stage II periodontitis if clinical attachment loss reached 3-4 mm [15]. Patients with systemic diseases, pregnant and lactating females, smokers and tobacco chewers, patients who are not compliant with oral hygiene procedures, or those with a history of antibiotic and periodontal therapy in the last three months were excluded from our study.

The study protocol was approved by the Research Ethical Committee of the Faculty of Dentistry, Mansoura University (approval number: A12020822). The patients were informed about the treatment that they received and the steps that have been taken. This includes the possible beneficial effects or risks and other treatment options according to the rules of the ethical committee. Written consent has been taken from each patient before performing any steps.

Sample size calculation

Estimation of sample size was based on probing pocket depth (PPD) index change after treatment as a clinical indicator for the effect of EPO injectable hydrogel in the treatment of stage II periodontitis patients as an adjunct to SRP retrieved from previous research [16]. Using G*Power Version 3.0.10 to calculate sample size based on the effect size of 1.05, two-tailed test, α error of 0.05, and power of 90%, the total sample size was 80 patients (20 patients in each group).

Randomization

Using computer-generated sequencing, the selected patients were randomly allocated into two equal groups. It was a single-blind study, where the examiner was the same practitioner. Patients were assigned before the SRP as it is considered a treatment option itself. Patients in the control group received SRP, while those in the study group received SRP followed by injectable hydrogel containing EPO.

Methods

Intervention

Current research included two groups: control and study groups. The control group received SRP only, while those in the study group received SRP followed by injectable hydrogel containing EPO.

Phase I periodontal therapy has been carried out for patients in both groups entailing SRP with ultrasonic tips and Gracey curettes, and oral hygiene instructions will be given to patients.

After the completion of SRP, for patients in the study groups, the hydrogel was injected once every two weeks for two months. Patients were instructed to evade hard food biting to avoid soft tissue traumatization and not to brush, floss, or use interproximal cleaning aids in the treated areas for the next 12 hours [17]. 

Hydrogel Application

Subgingival application of the hydrogel was delivered through a 3 ml disposable syringe with a blunt needle bent at its shank. The hydrogel was injected immediately after SRP. The application was performed until the pocket was filled and excess hydrogel was displayed. In the recall sessions, any side effects were assessed, and any supragingival deposits were removed. No chemotherapeutic agents were prescribed after treatment [18].

Periodontal Assessment

Clinical parameters including plaque index (PI), gingival index (GI), PPD, and clinical attachment level (CAL) were assessed at baseline and two months post treatment [19-22].

Gingival Crevicular Fluid (GCF) Sample Collection

Samples were collected from all subjects in both groups at baseline and two months after periodontal therapy (either control "SRP" or study group "EPO"). Isolation of the target site was performed with cotton rolls and dried by a gentle air stream to avoid contamination with saliva. Before sample collection, any supragingival deposits were removed with cotton pellets. Using a sterile tweezer, one paper point (Paperstrip, Oraflow Inc., Smithtown, New York, United States) was inserted into the selected pocket till feeling light resistance and left in situ for 30 seconds. Care was taken during GCF sampling to avoid periodontal traumatization. Blood- or saliva-contaminated samples were discarded. Paper points were placed in sterile Eppendorf tubes containing 100 μl phosphate buffer pH 7.4 and stored at -20°C till the time of analysis [23]. 

GCF samples were analyzed to assess the level of IL-1β and osteocalcin using commercially available enzyme-linked immunosorbent assay (ELISA) kits (Catalog No. E0143 Hu and No. E1555Hu, respectively) by following the manufacturer instructions [23]​.

Statistical analyses

The statistical analysis was performed to assess and compare the difference between two treatment groups, the control group (SRP) and the study group (EPO), at different times of observation (T0=baseline and T1=after two months); two-way repeated measure analysis of variance (rep-ANOVA) is proposed or corresponding statistical analysis for nonparametric data at 0.05 level.

The data were gathered, verified, amended, and structured in tabular and graphical formats utilizing Microsoft Excel 2016. The data underwent outlier detection and management utilizing IBM SPSS Statistics for macOS, Version 29.0 (Released 2022; IBM Corp., Armonk, New York, United States). At the 0.05 level, normality tests were conducted to determine whether the data are parametric or nonparametric using the Shapiro-Wilk and/or Kolmogorov-Smirnov test. The results of both stability and in-use stability were normally distributed, i.e., parametric data, as revealed by the Shapiro-Wilk test. All statistical analyses were carried out using the computer software IBM SPSS Statistics for macOS, Version 29.0 (Released 2022; IBM Corp., Armonk, New York, United States) [24].

## Results

Clinical parameters

**Figure 1:**
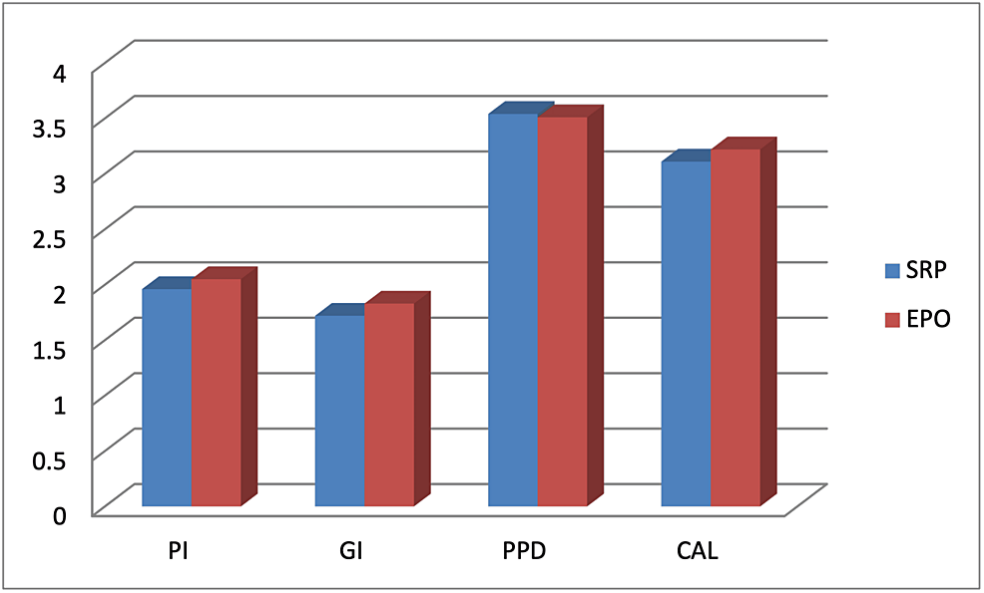
Comparisons between all tested clinical parameters detected between SRP and EPO groups. Data presented as average of 20 patients. No significant difference in all tested clinical parameters was detected between the SRP and EPO groups PI: plaque index; GI: gingival index; PPD: probing depth; CAL: clinical attachment loss; SRP: scaling and root planing; EPO: erythropoietin

At baseline, there were no significant differences between the control and study groups in all tested clinical parameters (p>0.05) (Table 1, Figure 1).

**Table 1:** Comparison of osteocalcin and IL-1ꞵ GCF levels between the study and control groups before and after treatment. Data presented in terms of mean and standard deviation. Means followed by different letters are significantly different according to DMRTs. At 0.05 level *, **, and *** significant at p<0.05, p<0.01, and p<0.001; ns non-significant at p>0.05 PI: plaque index; GI: gingival index; PPD: probing pocket depth; CAL: clinical attachment level; SRP: scaling and root planing; EPO: erythropoietin; ANOVA: analysis of variance; DMRT: Duncan's Multiple Range Test

	PI		GI		PPD		CAL	
	SRP (control)	EPO (study)	SRP (control)	EPO (study)	SRP (control)	EPO (study)	SRP (control)	EPO (study)
Baseline	1.96±0.14 a	2.05±0.30 a	1.72±0.23 b	1.83±0.26 b	3.54±0.27 c	3.51±0.23 c	3.11±0.40 d	3.22±0.35 d
Two months	2.98±0.27 e	2.78±0.22 e	1.34±0.16 f	1.13±0.22 g	2.98±0.27 h	2.78±0.22 i	2.68±0.37 j	2.36±0.40 k
Paired t-test	<0.001***	<0.001***	<0.001***	<0.001***	<0.001***	<0.001***	<0.001***	<0.001***
Two-way repeated measure ANOVA								
Groups	<0.001***							
Time	<0.001***							
Group×time	<0.001***							

**Figure 2:**
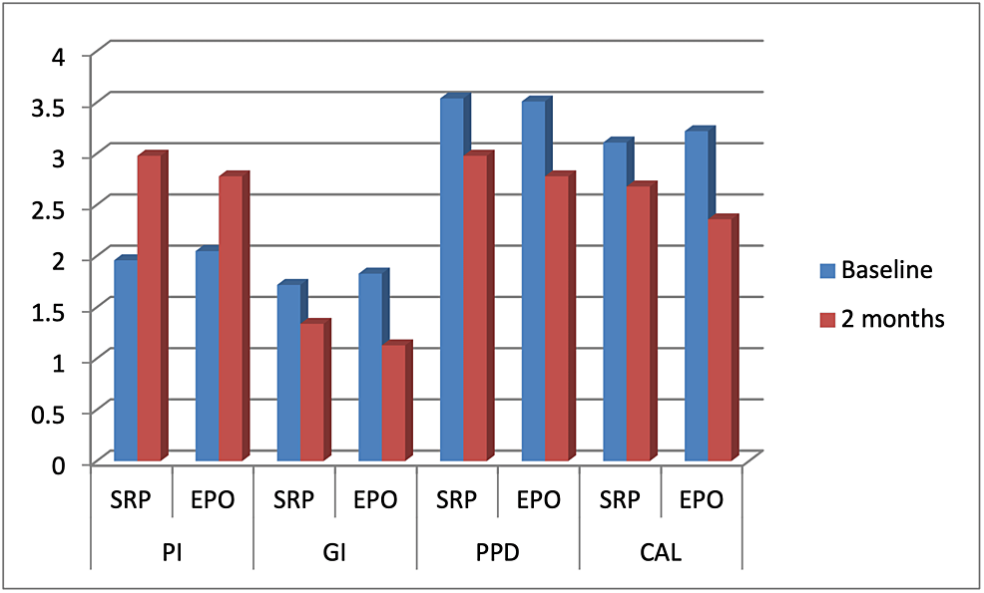
Comparison of clinical indices between the study and control groups before and after treatment. Bars present the average of 20 patients. PI: plaque index; GI: gingival index; PPD: probing depth; CAL: clinical attachment loss; SRP: scaling and root planing; EPO: erythropoietin

Two months post treatment, the PI scores decreased significantly in both groups from 2±0.09 and 2.04±0.31 to 1.3±0.21 and 1.363±0.24 in the control and study groups, respectively (p<0.001). However, the difference between the two groups was non-significant (p=0.38) (Table 2, Table 3, Figure 2).

**Table 2:** Comparison of clinical indices between the study and control groups before treatment. Data of clinical indices presented as mean and standard deviation. *, **, and *** significant at p<0.05, p<0.01, and p<0.001; ns non-significant at p>0.05 PI: plaque index; GI: gingival index; PPD: probing depth; CAL: clinical attachment loss; SRP: scaling and root planing; EPO: erythropoietin

Baseline	SRP	EPO	Independent t-test
			p-value
PI	1.96±0.14	2.05±0.30	0.120 ns
GI	1.72±0.23	1.83±0.26	0.272 ns
PPD	3.54±0.27	3.51±0.23	0.89 ns
CAL	3.11±0.40	3.22±0.35	0.349 ns

**Table 3:** Comparison of clinical indices between the study and control groups after two-month follow-up. Data is presented as mean and standard deviations. *, **, and *** significant at p<0.05, p<0.01, and p<0.001; ns non-significant at p>0.05 PI: plaque index; GI: gingival index; PPD: probing depth; CAL: clinical attachment loss; SRP: scaling and root planing; EPO: erythropoietin

Two months	SRP (control group)	EPO (study group)	Independent sample t-test
			p-value
PI	2.98±0.27	2.78±0.22	0.831 ns
GI	1.34±0.16	1.13±0.22	0.002 **
PPD	2.98±0.27	2.78±0.22	<0.001***
CAL	2.68±0.37	2.36±0.40	<0.001***

At two-month recall visit, the GI scores decreased significantly in both groups from 1.722±0.24 and 1.8±0.27 to 1.3 ±0.16 and 1.12±0.22 in the control and study groups, respectively (p<0.001). The inter-group comparison revealed a significant difference between the control and study groups (p<0.001), with more favorable results in the study group (Table 2, Table 3, Figure 2).

At two months, the PPD mean values showed a statistically significant reduction from 3.45±0.29 and 3.51±0.24 to 2.98±0.28 and 2.775±0.23 in the control and study groups, respectively (p<0.001). A significant difference was detected between the two groups (p<0.001) (Table 2, Table 3, Figure 2).

Two months post treatment, there was a statistically significant reduction of CAL from 3.11±0.43 and 3.22±0.38 to 2.679±0.4 and 2.355±0.44 in the control and study groups, respectively (p<0.001). The comparison between the two groups showed a statistically significant difference (p<0.001) (Table 2, Table 3, Figure 2).

Laboratory Assays

IL-1ꞵ Level

**Figure 3:**
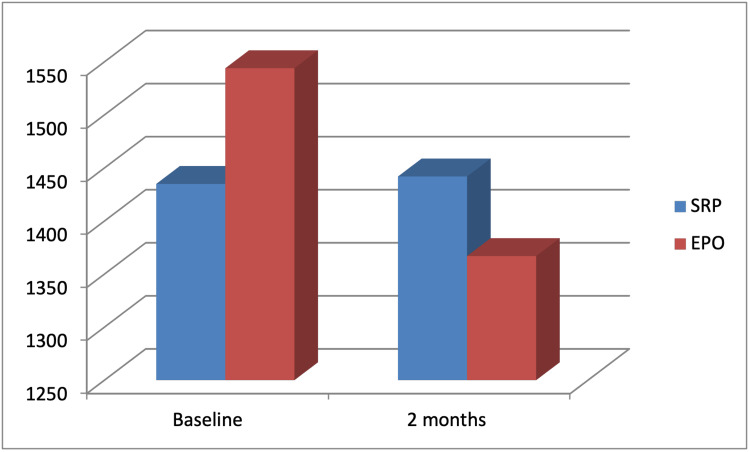
Comparison of osteocalcin GCF level between the study and control groups before and after treatment. Data presented as average of 20 patients. SRP: scaling and root planing; EPO: erythropoietin; GCF: gingival crevicular fluid

IL-1ꞵ level decreased in the study group from 1544±156 at baseline to 1384±231 at two-month recall visit. A significant difference was detected in the study group between baseline and after two months (p<0.001). However, IL-1ꞵ level decreased in the control group from 1442±102 to 1435±176 at two months. There was no significant difference between baseline and two months post treatment (p=0.855) (Table 4, Figure 3).

**Table 4:** Comparison of clinical indices between the study and control groups before and after treatment. Data is presented as average and standard deviation of 20 patients. Means followed by different letters are significantly different according to DMRTs. At 0.05 level *, **, and *** significant at p<0.05, p<0.01, and p<0.001; ns non-significant at p>0.05 PI: plaque index; GI: gingival index; PPD: probing pocket depth; CAL: clinical attachment level; SRP: scaling and root planing; EPO: erythropoietin; ANOVA: analysis of variance; DMRT: Duncan's Multiple Range Test

	PI		GI		PPD		CAL	
	SRP (control)	EPO (study)	SRP (control)	EPO (study)	SRP (control)	EPO (study)	SRP (control)	EPO (study)
Baseline	1.96±0.14 a	2.05±0.30 a	1.72±0.23 b	1.83±0.26 b	3.54±0.27 c	3.51±0.23 c	3.11±0.40 d	3.22±0.35 d
Two months	2.98±0.27 e	2.78±0.22 e	1.34±0.16 f	1.13±0.22 g	2.98±0.27 h	2.78±0.22 i	2.68±0.37 j	2.36±0.40 k
Paired t-test	<0.001***	<0.001***	<0.001***	<0.001***	<0.001***	<0.001***	<0.001***	<0.001***
Two-way repeated measure ANOVA								
Groups	<0.001***							
Time	<0.001***							
Group×time	<0.001***							

Osteocalcin Level

**Figure 4:**
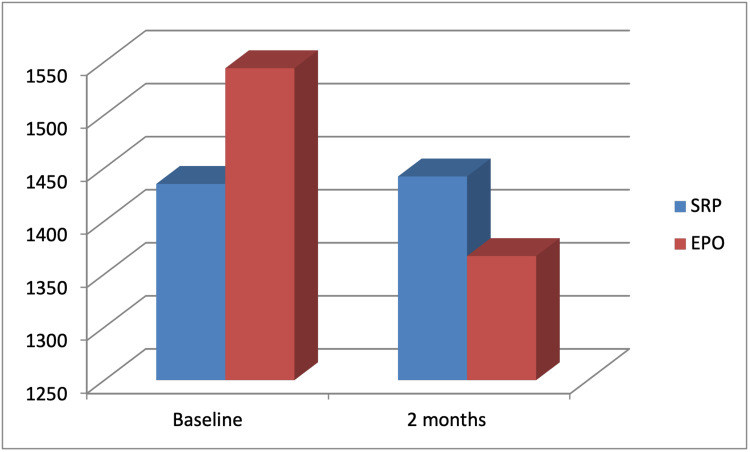
Comparison of IL-1ꞵ GCF level between the study and control groups before and after treatment. Data presented as average of 20 patients. SRP: scaling and root planing; EPO: erythropoietin; IL: interleukin

Osteocalcin level changed in the study group from 50.6±4 at baseline to 57.2±14.8 at two-month recall visit. A significant difference was detected between baseline and after two months (p<0.001). Also, osteocalcin level decreased in the control group from 62±9.6 at baseline to 57.6±9.1 at two months. There was a significant difference between baseline and two months post treatment (p<0.001) (Table 4, Figure 4).

## Discussion

Although eliminating the bacterial load by NSPT from the inflamed periodontal pockets is the primary goal in periodontal therapy, the need for adjunctive therapy by either systemic or local routes to improve the treatment outcomes become mandatory in many cases and has proven to have additional successful results [2]. 

Emerging from the dramatic role of the host immune-inflammatory response to the colonizing periodontopathic bacteria and the subsequent mechanisms of periodontal tissue destruction in the development of periodontitis, the need for host modulation has been raised. Thus, in association with the mechanical debridement of the periodontal pocket, the adjunctive use of a host immune-modulatory agent became an inevitable consequence [25,26].

Fulfilling these two therapeutic targets in the form of elimination of the bacterial load and modulation of the host immune-inflammatory response, this study was designed to investigate the efficacy of local EPO injectable hydrogel as an adjunct to SRP in the treatment of stage II periodontitis patient. This study was designed as a randomized, single-blind study. The randomization was done through computer-generated randomizing tables to avoid bias [27].

The hydrogel biocompatibility and stable structure (for 14 days) were validated. The used therapeutic agent was loaded on hydrogel formula permitting the prolonged drug action in the periodontal pocket, as this formula resisted the washing by the gingival fluid. Based on that, the intra-pocket application of the drug was performed every two weeks minimizing the total dose of the used drug as well as providing protection for the patient from the suggested medication side effects, via prevention of the passage of the used drug into the bloodstream [28,29]. Furthermore, delivering the therapeutic agent by the delivery system in nano-sized particles allows it to become in intimate contact with the irregular surface topography of the periodontal pockets [30].

Regarding the present study, at baseline, there was no statistically significant difference in all tested clinical parameters (PPD, CAL, GI, and PI) between the control and study groups ensuring unbiased comparable treatment outcomes during the follow-up intervals.

Regarding PI, two months post treatment, both groups showed significant improvement. These results ensured the successful performance role of the operator in the meticulous phase I therapy, patient motivation, and ensured the patient's commitment to oral hygiene instructions as they were maintained under a stringent program. However, the inter-group statistical analysis showed no significant difference [31].

In the present study, there was a significantly improved GI in both tested groups, which could be explained by the successful role of phase I therapy in minimizing the inflammatory cascade [2]. The superior significant improvement in GI noticed in the study group shifts the insight to the anti-inflammatory effect of EPO. This could be attributed to the effect of EPO in decreasing the release of the pro-inflammatory cytokines along with promoting the production of the anti-inflammatory agents, which interrupts the continuous activation of the immune response and, subsequently, shifts the microenvironment a little bit toward hemostasis [7]. Similar results were observed by Aslroosta et al. [32].

The decrease in PPD and CAL are the major clinical outcomes to ascertain the success of any periodontal therapy. PPD and CAL decreased significantly within both treated groups compared to baseline. This finding could be explained by the logical reduction in gingival inflammation secondary to minimizing the microbial load which promotes the healing of the tissues with a resultant decrease in both PPD and CAL [33].

Moreover, there was a statistically significant difference between both tested groups. This could be attributed to more than one factor. The combined positive effect was obtained from the meticulous conventional treatment performed by the operator which succeeded in minimizing the inflammation and the obtained additional EPO anti-inflammatory effects that create a microenvironment favorable for tissue healing [7].

Regarding the significant clinical improvement following SRP, this study's results are by Komala et al. and Megavath et al. They evaluated the clinical effect following SRP and reported a significant improvement in all tested clinical parameters [34,35].

To the best of our knowledge, to date, there a very few studies carried out on the clinical effect of EPO in the treatment of periodontitis. By this study results, Aslroosta et al. [32] investigated the effect of using EPO along with non-surgical treatment. They reported a significant improvement in GI, CAL, and PPD compared to the conventionally treated group [32].

The present study detected no statistically significant reduction in the control group in the level of IL-1ꞵ. This finding agrees with Al‐Shammari et al. and Aljateeli et al., results which found no significant difference in GCF IL-1β levels after SRP. These results may suggest a prolonged production of certain pro-inflammatory cytokines even after phase I therapy that needs extended periods of oral hygiene maintenance to let a chance for the inflammatory state to subside or resolute [36,37]. However, the present study findings disagree with Bıyıkoğlu et al. and Cicek et al., results who reported decreased levels of GCF IL-1ꞵ after SRP [38,39].

Regarding the GCF osteocalcin level, the present study revealed a significantly decreased osteocalcin level in both groups. This finding coincides with Moussa [40], Cutando et al. [41], and AbdAllah et al. [42] which informed a significant reduction in GCF osteocalcin levels after NSPT. In contrast, these results disagree with the study of Hakobyan et al. [43], who reported that GCF osteocalcin level was elevated. They added that a negative correlation was found between GCF osteocalcin level and the tested clinical parameters.

Limitations of the study

We don't include a healthy group as a negative control group, as this group of healthy individuals will be needed only for the assessment of the level of GCF markers tested among those healthy individuals which was previously determined in much previous research, from one side, and, from the other side, it is not one of the targets of this study. Additionally, the age distribution of the patients in both periodontitis groups was slightly older than that of the healthy group, which could be explained by the fact that periodontitis naturally progresses with age. 

## Conclusions

This preliminary study revealed that EPO can be considered an effective periodontal therapeutic modality as an adjunctive to SRP. Further study is required to confirm our study's promising therapeutic findings and its possible clinical application as these observations may provide an auspicious approach to reducing the need for invasive approaches that cause more patient discomfort, thereby highlighting novel therapeutic targets for periodontitis treatment.
